# LAMB3 mediates apoptotic, proliferative, invasive, and metastatic behaviors in pancreatic cancer by regulating the PI3K/Akt signaling pathway

**DOI:** 10.1038/s41419-019-1320-z

**Published:** 2019-03-08

**Authors:** Hong Zhang, Yao-zhen Pan, May Cheung, Mary Cao, Chao Yu, Ling Chen, Lei Zhan, Zhi-wei He, Cheng-yi Sun

**Affiliations:** 1 Guizhou Medical University, Guiyang, Guizhou China; 2 Department of Hepatobiliary Surgery, The Affiliated Hospital of Guizhou Medical University, Guiyang, Guizhou China; 3 Key Laboratory of Hepatobiliary-Pancreas-Spleen Surgery of Guizhou Medical University, Guiyang, Guizhou China; 4 Ontario Cancer Institute, Princess Margaret Cancer Centre, University of Toronto, Toronto, ON Canada

## Abstract

The poor prognosis of patients with pancreatic ductal adenocarcinoma (PDAC) is partially attributed to the invasive and metastatic behavior of this disease. Laminin subunit beta-3 (LAMB3) encodes one of the three subunits of LM-332, an extracellular matrix protein secreted by cultured human keratinocytes. In addition, LAMB3 is involved in the invasive and metastatic abilities of some types of cancer, including colon, pancreas, lung, cervix, stomach, and prostate cancer, but the role and mechanism of LAMB3 in PDAC have not been previously determined. Herein, we tentatively investigated the role of LAMB3 in the malignant biological behavior of PDAC. In this study, we demonstrated that LAMB3 is upregulated in PDAC. Inhibition of LAMB3 abrogated the tumorigenic outcomes of PI3K/Akt signaling pathway activation, including those involving cell cycle arrest, cell apoptosis, proliferation, invasion and migration in vitro, and tumor growth and liver metastasis in vivo. Our results showed that LAMB3 could mediate cell cycle arrest and apoptosis in PDAC cells and alter the proliferative, invasive, and metastatic behaviors of PDAC by regulating the PI3K/Akt signaling pathway. LAMB3 may be a novel therapeutic target for the treatment of PDAC in the future.

## Introduction

Pancreatic ductal adenocarcinoma (PDAC) is a malignant tumor that is difficult to diagnose early and treat worldwide^1^. Despite improvements in treatment, patient prognosis remains poor, partly attributed to devastating local tumor invasion and distant metastasis^2,3^. PDAC develops as a result of genetic and epigenetic alterations. Therefore, obtaining a better understanding of the potential mechanisms for the growth, metastasis, apoptosis, and tumorigenic properties of PDAC will provide opportunities for the development of new therapeutic strategies for this disease^4,5^. We analyzed expression profiles in The Cancer Genome Atlas (TCGA) and Gene Expression Omnibus (GEO) databases and found laminin subunit beta-3 (LAMB3) to be upregulated in PDAC^6^. We investigated LAMB3 expression in PDAC using 102 matched pairs of PDAC and adjacent normal pancreatic tissues with a tissue microarray (TMA) and found that the product of this gene, which encodes a member of the kinesin protein family, may play a vital role in PDAC carcinogenesis.

Laminins are important and biologically active components of the basal lamina that influence cell differentiation, migration, adhesion, proliferation, and survival^7,8^. LAMB3 encodes one of the three subunits of LM-332, an extracellular matrix protein secreted by cultured human keratinocytes. While LAMB3 is involved in the invasive and metastatic abilities of some types of cancer, including colon, pancreas, lung, cervix, stomach, and prostate cancer, its mechanism of action in pancreatic cancer has not been investigated previously^9–11^.

Phosphatidylinositol 3-kinase (PI3K) and protein kinase B (PKB/Akt) are the key proteins in the PI3K/Akt signaling pathway. This pathway is regulated by multiple mechanisms and is involved in numerous types of cancer^12–14^. Activated Akt is involved in regulating cell cycle and the proliferative, antiapoptotic, metastatic, and invasive abilities of cancer cells^15–17^. Phosphoinositide‑dependent kinase 1 (PDK1) partially activates Akt via phosphorylation of T308, and phosphorylation of S473 by PDK2 is needed for full activation^18,19^.

Our results show that LAMB3 is upregulated in PDAC, and suppressing its expression reduces cell proliferation, invasion, and migration by downregulating epithelial‒mesenchymal transition (EMT)-related proteins (N-cadherin, vimentin, Snail, Slug). LAMB3 suppression also significantly decreases Akt phosphorylation and inhibits the transcription of PI3K, reducing its activation. These results suggest that LAMB3 promotes tumor invasion via Akt activation through the PI3K axis in PDAC cells. Our findings identified a novel molecular mechanism of action for LAMB3 in PDAC, potentially suggesting a novel therapeutic strategy for blocking PDAC invasion and metastasis.

## Results

### LAMB3 is positively correlated with PDAC development

**Fig. 1:**
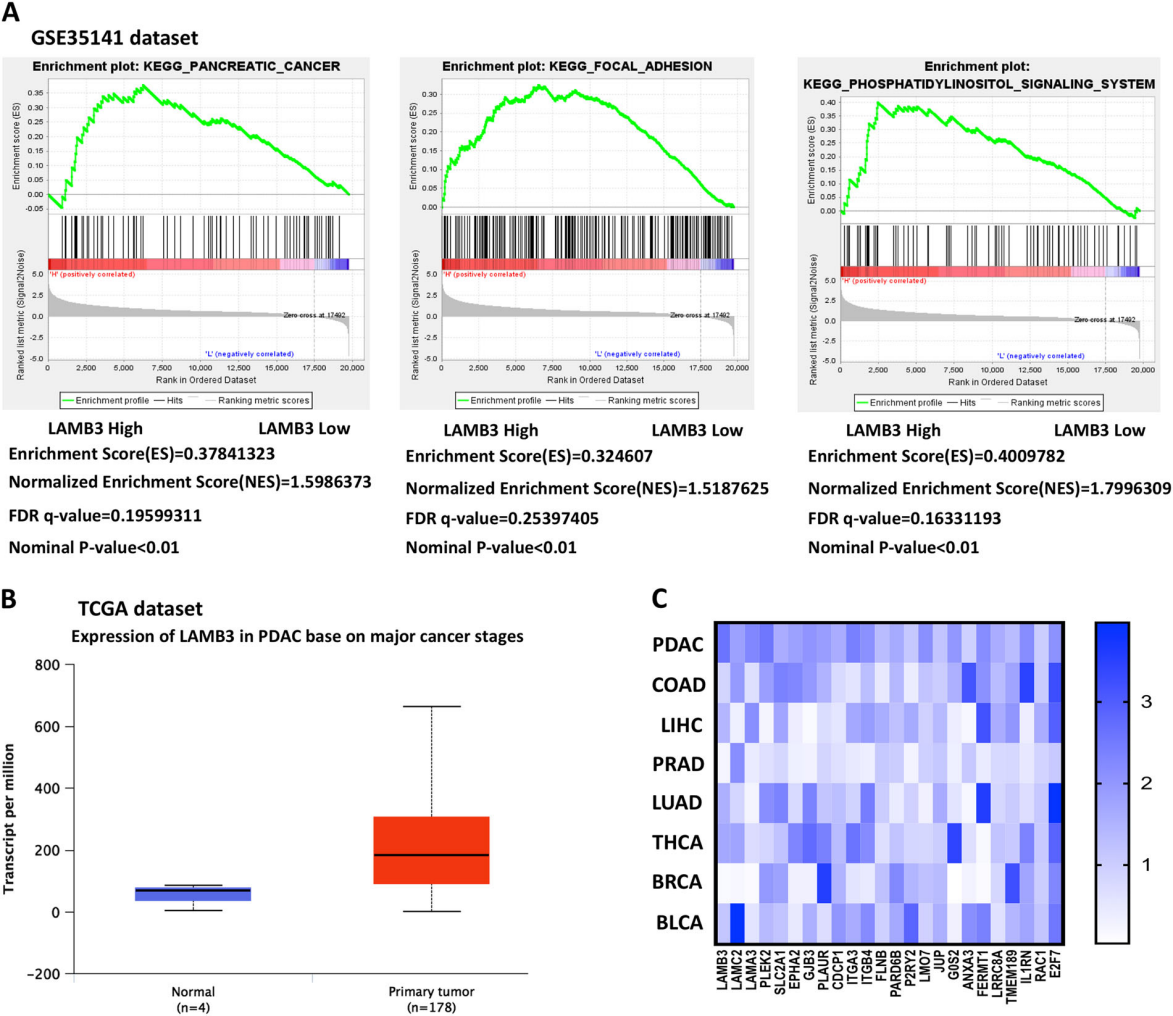
LAMB3 is positively correlated with pancreatic ductal adenocarcinoma (PDAC) development. **a** The gene set enrichment analysis (GSEA) plot indicated that high LAMB3 expression was significantly positively correlated with PDAC. **b** The Cancer Genome Atlas (TCGA) dataset suggested that LAMB3 was correlated with PDAC disease stage. **c** Heat map showing the relative expression of LAMB3 and genes positively correlated with LAMB3 in eight common solid tumors

Through analyzing published messenger RNA (mRNA) expression profiles in the NCBI GEO (GSE35141; https://www.ncbi.nlm.nih.gov/geo/), we found that LAMB3 mRNA was significantly upregulated in PDAC tissues compared with normal pancreas tissues. The gene set enrichment analysis (GSEA) plot indicated that high LAMB3 expression was significantly positively correlated with PDAC adhesion and migration by activating the phosphatidylinositol signaling system (Fig. 1a). The TCGA dataset showed that LAMB3 upregulation was associated with PDAC disease stage (Fig. 1b). The heat map showed the relative median expression of LAMB3 and genes positively correlated with LAMB3 in eight common solid tumors, PAAD (pancreatic adenocarcinoma), COAD (colon adenocarcinoma), LIHC (liver hepatocellular carcinoma), PRAD (prostate adenocarcinoma), LUAD (lung adenocarcinoma), THCA (thyroid carcinoma), BRCA (invasive breast carcinoma), and BLCA (bladder urothelial carcinoma), based on datasets from the TCGA database; LAMB3 was positively correlated with PDAC (Fig. 1c).

### LAMB3 expression is increased in PDAC tissues

**Fig. 2:**
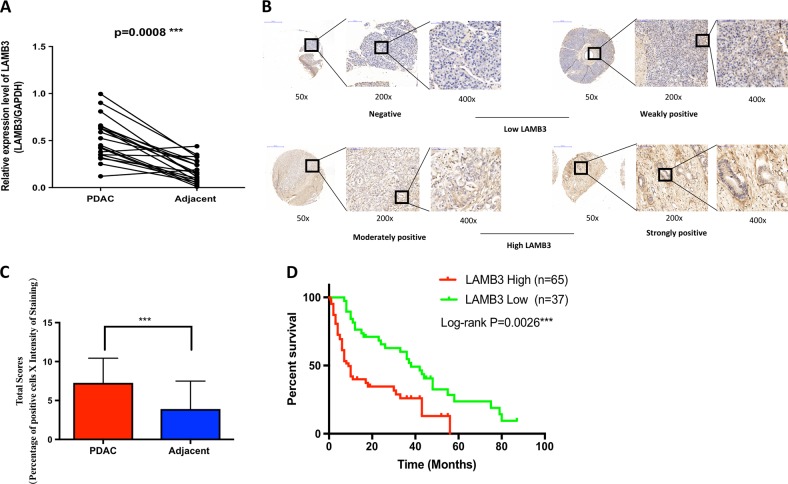
LAMB3 expression is increased in pancreatic ductal adenocarcinoma (PDAC) tissues. **a** LAMB3 mRNA expression levels in PDAC and adjacent normal tissues (*n* = 20, ***P < 0.001). **b** Representative images of immunohistochemistry (IHC) staining for LAMB3 magnified 50-fold, 200-fold, and 400-fold in tissue microarrays (TMAs) (scale bars: 500, 100, and 50 μm from left to right). **c** Comparison of overall scores for the LAMB3 assessment on TMA slides, ***P < 0.001. **d** The overall survival rate of patients with PDAC was evaluated based on LAMB3 expression using Kaplan–Meier analysis (*n* = 102, **P = 0.0026)

We analyzed LAMB3 mRNA expression levels in 20 paired patient samples of PDAC and adjacent normal tissues. LAMB3 expression was obviously increased in PDAC tissues compared with normal tissues (Fig. 2a). We confirmed this finding through TMAs containing 102 matched pairs of PDAC and adjacent normal pancreas tissues (Fig. 2b). According to the total immunohistochemistry (IHC) score (percentage of positive cells x staining intensity), LAMB3 was expressed at higher levels in PDAC tumor tissues than in adjacent normal tissues (Fig. 2c). Moreover, an analysis of the clinical characteristics of PDAC revealed that high LAMB3 expression was related to more advanced tumor node metastasis (TNM) stage (Supplementary Table 1). Furthermore, Kaplan–Meier survival curves revealed an obvious correlation between high LAMB3 expression and poor prognosis of patients with PDAC (*P* = 0.0026; Fig. 2d).

### LAMB3 is expressed in PDAC cells and promotes PDAC cell proliferation in vitro

**Fig. 3:**
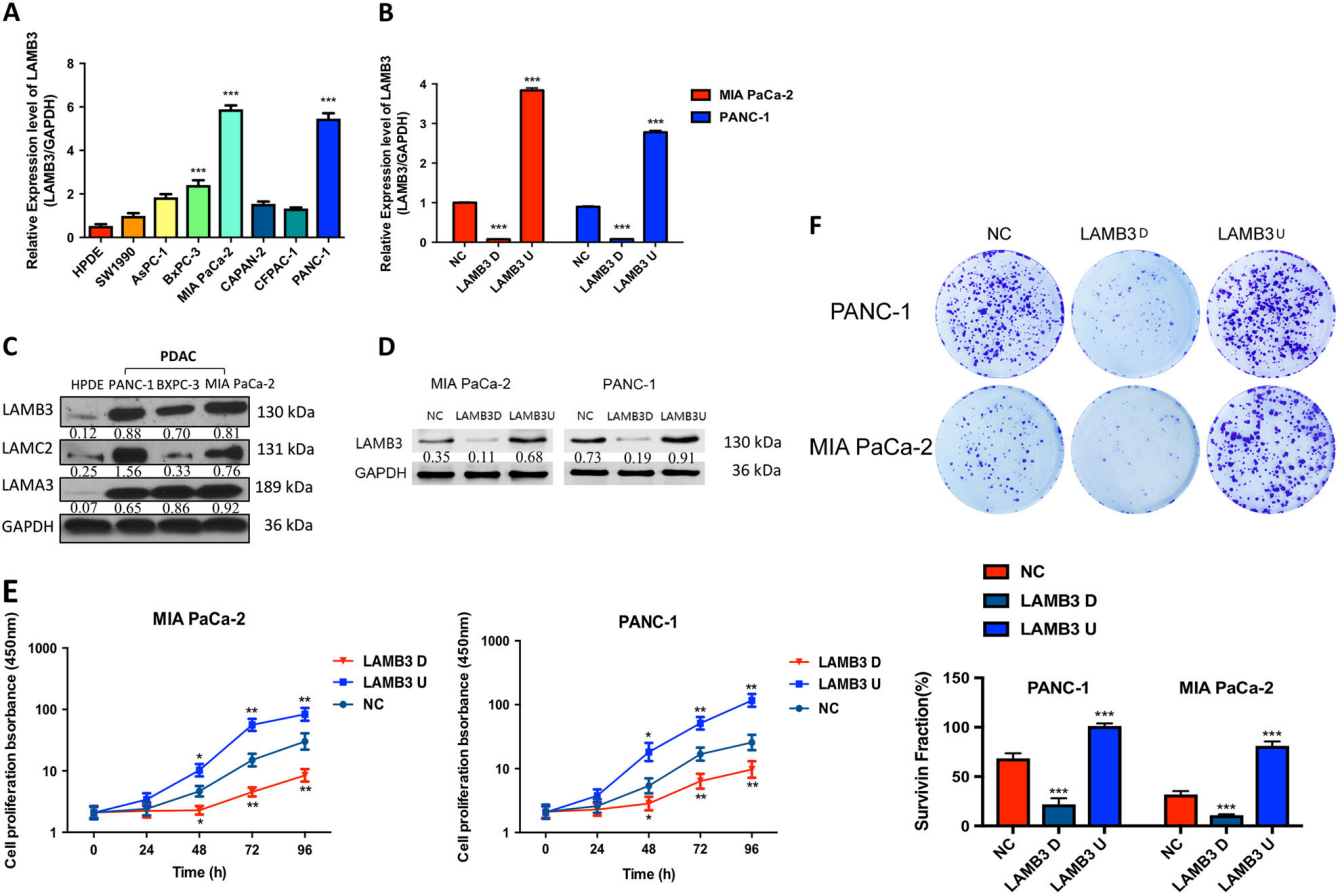
LAMB3 is expressed in pancreatic ductal adenocarcinoma (PDAC) cells and promotes PDAC cell proliferation in vitro. **a** LAMB3 mRNA expression levels in a normal pancreatic cell line and seven different established PDAC cell lines. **b** LAMB3 mRNA expression was examined by quantitative PCR (qPCR) in PANC-1 and MIA PaCa-2 cells stably transfected with overexpressing LAMB3 overexpression (LAMB3U), LAMB3 knockdown (LAMB3D), or empty vector plasmids (NC). **c** Cell lysates were prepared from a normal pancreatic cell line and three pancreatic cancer cell lines (PANC-1, BXPC-3, and MIA PaCa-2) and examined by western blot analysis. **d** LAMB3 protein levels were examined by western blot analysis in transfected PANC-1 and MIA PaCa-2 cells. **e** Cell proliferation was compared using CCK-8 assays in the LAMB3D, LAMB3U, and NC groups. **f** Colony formation assays were performed in the LAMB3D, LAMB3U, and NC groups. Each figure is representative of three independent experiments

We investigated LAMB3 mRNA levels in seven PDAC cell lines and human pancreatic ductal epithelial (HPDE) cells (Fig. 3a). LAMB3 expression levels were upregulated in the seven PDAC cell lines compared with the HPDE cells. Then, we chose three cell lines with the highest LAMB3 protein expression levels to evaluate LAMB3 expression after cell manipulation (Fig. 3c). LAMB3 expression was obviously increased in PANC-1 and MIA PaCa-2 cells at both the mRNA and protein levels. We also examined LAMA3 and LAMC2 protein expression and found it to be increased in the PDAC cell lines compared to the normal pancreatic cell line. We used a lentiviral delivery system to generate MIA PaCa-2 cells and PANC-1 cells stably overexpressing LAMB3 (LAMB3U) or with stable LAMB3 knockdown (LAMB3D). To confirm LAMB3 overexpression and silencing, we compared LAMB3U and LAMB3D cells with the corresponding negative control (NC) cells by quantitative PCR (qPCR) and western blotting. GAPDH was used as the loading control (Fig. 3b–d). Cell counting kit-8 (CCK-8) and colony formation assays suggested that PDAC proliferation was inhibited by LAMB3 knockdown and increased by LAMB3 overexpression (Fig. 3e).

### LAMB3 promotes PDAC cell migration and invasion in vitro

**Fig. 4:**
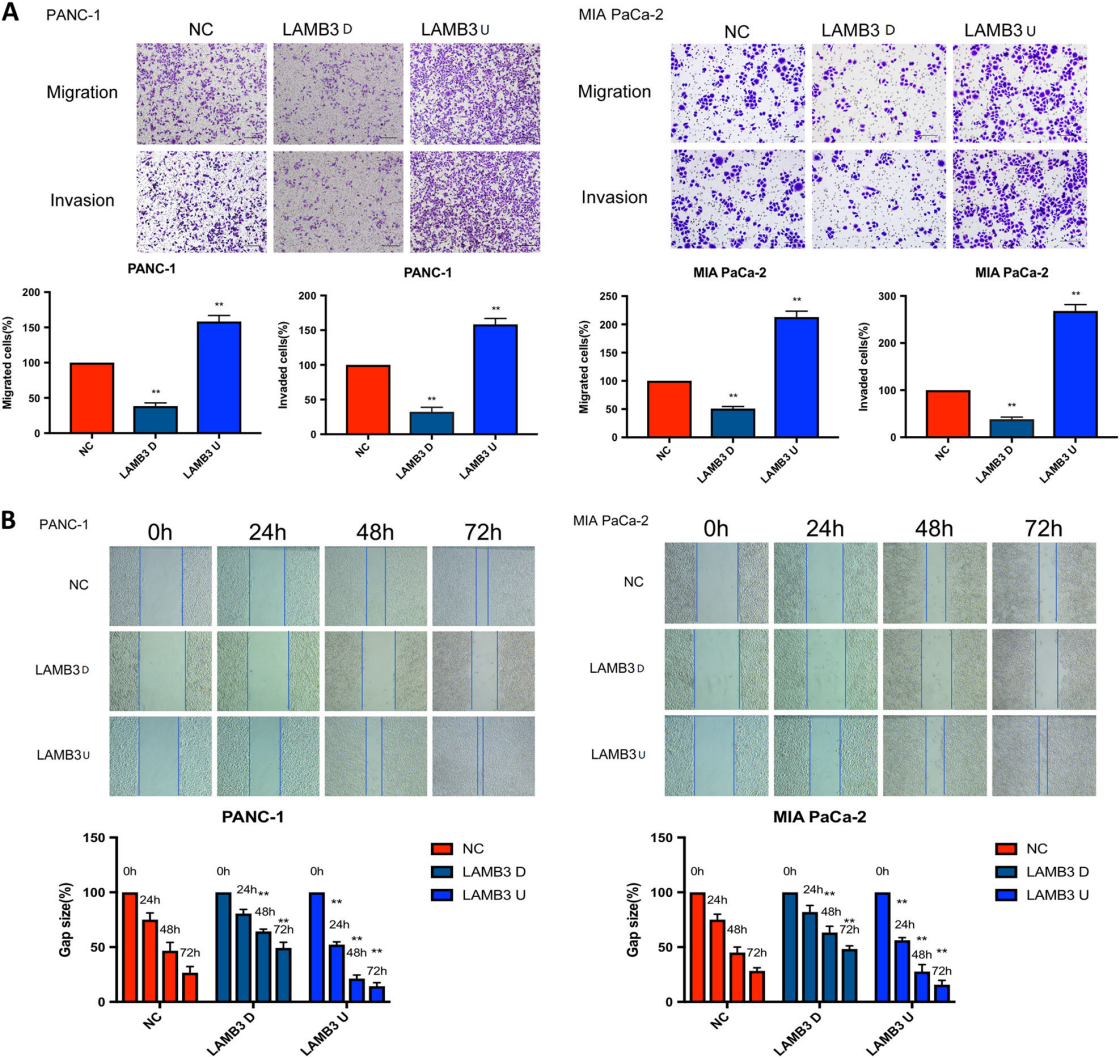
LAMB3 promotes pancreatic ductal adenocarcinoma (PDAC) cell migration and invasion in vitro. **a** LAMB3 knockdown suppressed both cell invasion and migration in wound healing assays and **b** transwell invasion and migration assays; the opposite results were observed in the LAMB3 overexpression group (**P < 0.01)

As our results indicated that LAMB3 expression correlated with local invasion and metastasis, we investigated the effect of LAMB3 on the invasive and metastatic capacities of PDAC cells using transwell and wound healing assays. We evaluated the wound width at 0, 24, 48, and 72 h after virus infection in wound healing assays and the percentage of invasive and metastatic cells at 24 h after virus infection in transwell assays. Transwell and wound healing assays both revealed that PDAC cell invasion and migration in vitro was promoted by LAMB3 overexpression and inhibited by LAMB3 knockdown (Fig. 4a, b).

### LAMB3 affects apoptosis and the cell cycle distribution of PDAC cells in vitro

**Fig. 5:**
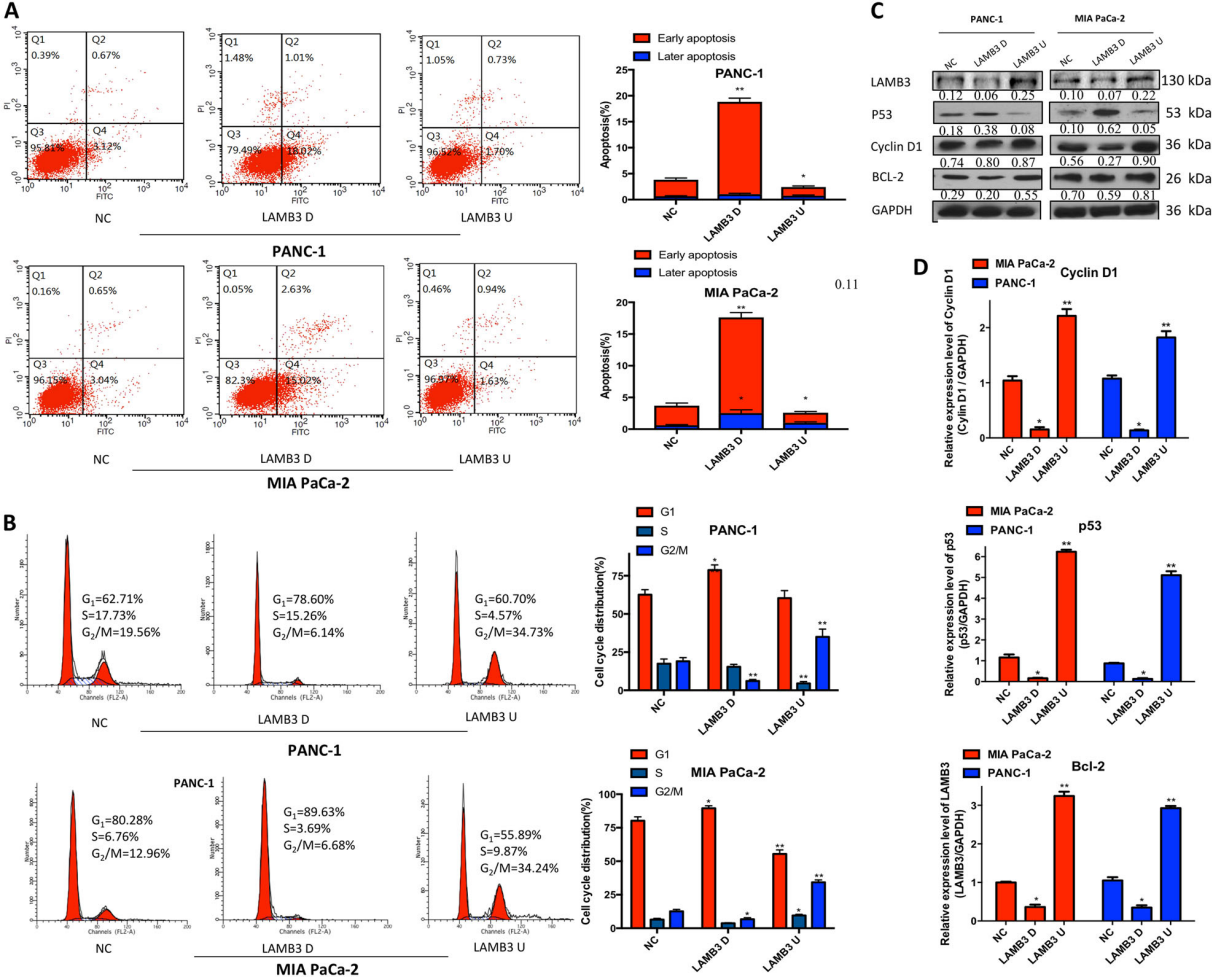
LAMB3 affects the survival and cell cycle of pancreatic cancer cell lines in vitro. **a** The percentage of apoptotic cells and **b** cell cycle changes were demonstrated by flow cytometry in both cell lines following the knockdown or overexpression of LAMB3 (*P < 0.05, **P < 0.01). **c** The protein and **d** mRNA expression levels of cell survival- and cell cycle-related proteins (cyclin D1, Bcl-2 and p53) were evaluated by western blot and quantitative PCR (qPCR) analyses in both cell lines following the knockdown and overexpression of LAMB3

Flow cytometry was used to explore whether LAMB3 promotes PDAC proliferation by regulating the cell cycle and/or apoptosis. Changes in LAMB3 levels affected the apoptotic ratio. In both cell lines with LAMB3 knockdown, the population of apoptotic cells increased, especially that of cells in early apoptosis. In both cell lines overexpressing LAMB3, only the number of early apoptotic cells decreased (Fig. 5a). Moreover, LAMB3 induced a dramatic alteration in cell cycle distribution. In both cell lines with LAMB3 knockdown compared with NC cells, the fraction of cells in G1 phase increased, while that of cells in S and G2/M phase decreased. Moreover, in both cell lines overexpressing LAMB3 compared with NC cells, the fraction of cells in G1 phase decreased, while that of cells in G2/M phase increased (Fig. 5b). Western blot and qPCR analyses showed that LAMB3 overexpression upregulated cyclin D1, which promoted cell cycle progression, and BCL-2, which inhibited apoptosis, and downregulated p53, which arrested cell cycle progression and promoted apoptosis; the LAMB3 knockdown group had the opposite effect (Fig. 5c, d).

### LAMB3 regulates PI3K-mediated Akt phosphorylation and EMT in PDAC cell lines

**Fig. 6:**
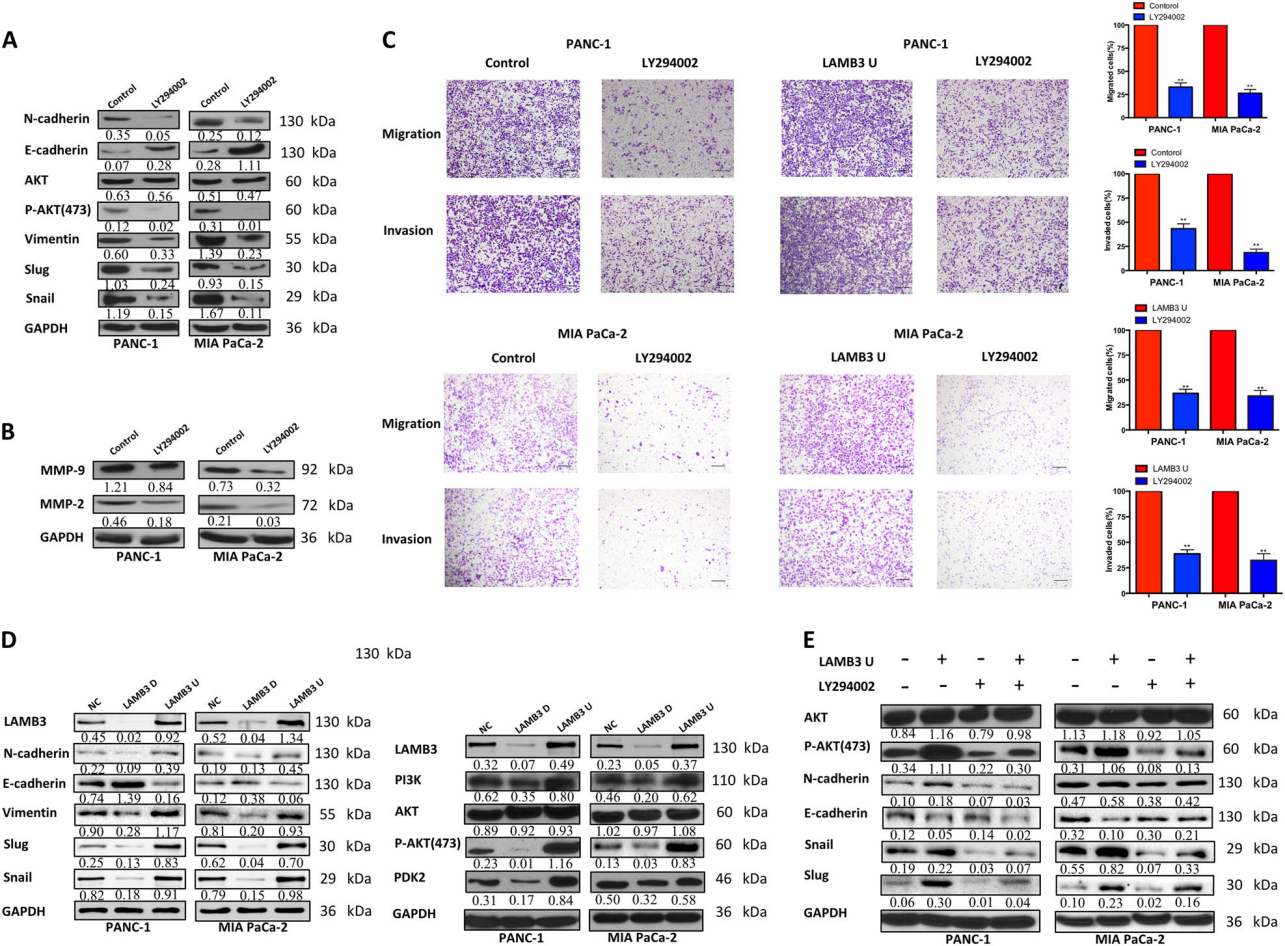
LAMB3 regulates PI3K-mediated Akt phosphorylation and epithelial‒mesenchymal transition (EMT) in pancreatic cells. **a** The levels of total Akt, p-Akt-S473, and the EMT-related proteins N-cadherin, E-cadherin, Snail, Slug, and vimentin were examined by western blot analysis, as were those of the **b** tumor invasion and migration-related proteins MMP2 and MMP9. **c** Invasion and migration were examined by transwell assays in control and LAMB3-overexpressing PANC-1 and MIA PaCa-2 cells after LY294002 (10 μM) treatment for 48 h. **d** EMT-related proteins and the PI3K/Akt-related proteins PI3K, PDK2, total Akt, and p-Akt-S473 were evaluated by western blot analysis in the LAMB3D, LAMB3U, and NC groups. **e** After treatment with or without 10 μM LY294002 for 48 h, the expression of EMT-related proteins, including N-cadherin, E-cadherin, Snail, Slug, Akt, and p-Akt-S473, was evaluated by western blot analysis in the LAMB3U and NC groups. Each figure is representative of three independent experiments

**Fig. 7:**
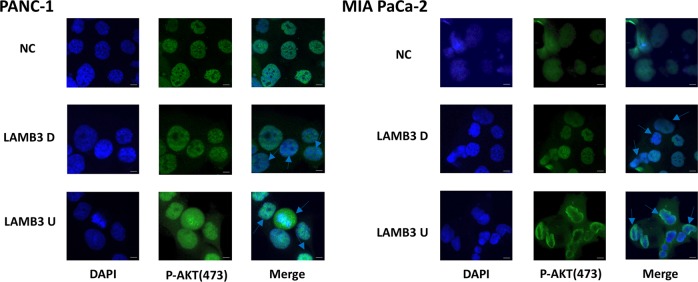
p-Akt expression by immunofluorescence analysis. Immunofluorescence analysis of p-Akt levels in the LAMB3U (bottom), LAMB3D (middle), and NC (top) groups in both cell lines (scale bar: 10 μm)

As mentioned above, our GSEA plot results indicated that high LAMB3 expression was significantly positively correlated with PDAC adhesion and migration via activation of the phosphatidylinositol signaling system. PI3K/Akt signaling appears to play an important role in PDAC progression. Inhibiting Akt with LY294002 decreased the protein levels of the EMT-related proteins vimentin, Slug, and Snail, and increased those of the epithelial marker E-cadherin (Fig. 6a). The protein expression levels of the tumor invasion and migration-related proteins matrix metalloproteinase 9 (MMP9) and MMP2 were also reduced by Akt inhibition (Fig. 6b). To elucidate whether inhibiting the PI3K/Akt signaling pathway can reduce PDAC cell invasion and migration, transwell invasion, and migration assays were performed using LY294002-treated PANC-1 and MIA PaCa-2 cells with or without LAMB3 overexpression. Inhibition of the PI3K/Akt signaling pathway significantly suppressed invasion and migration in both cell lines (Fig. 6c). Furthermore, we hypothesized that LAMB3 regulates cell migration and invasion via the PI3K/Akt signaling pathway. LAMB3 knockdown significantly decreased the levels of the EMT-related proteins Slug, Snail, vimentin, and N-cadherin, and increased E-cadherin levels; the levels of PI3K, PDK2, and phosphorylated Akt at S473 (p-Akt-S473) were also decreased, western blotting revealed that total Akt levels were not affected in either cell line, and LAMB3 overexpression had the opposite effect (Fig. 6d). We have shown that LAMB3 promotes PANC-1 and MIA PaCa-2 cell invasion and migration by transwell invasion and migration assays. To further verify whether these effects are mediated through the PI3K/Akt pathway at the protein level, NC and LAMB3-overexpressing PANC-1 and MIA PaCa-2 cells were treated with the Akt inhibitor LY294002, and the levels of Akt, p-Akt-S473 and the EMT-related proteins N-cadherin, E-cadherin, Snail, and Slug were examined by western blotting. LY294002 significantly decreased the levels of p-Akt-S473, N-cadherin, Snail, and Slug in both the NC and LAMB3-overexpressing groups, whereas E-cadherin levels were significantly increased in only LAMB3-overexpressing MIA PaCa-2 cells. The total levels of Akt were not significantly affected (Fig. 6e). Immunofluorescence staining was used to detect the expression and localization of p-Akt-S473 and to further verify the results presented above. PDAC cells with LAMB3 knockdown had decreased p-Akt-S473 levels, which correlated with an EMT phenotype, while the opposite results were observed in the LAMB3 overexpression group (Fig. 7).

### LAMB3 promotes pancreatic cancer cell proliferation in vivo

**Fig. 8:**
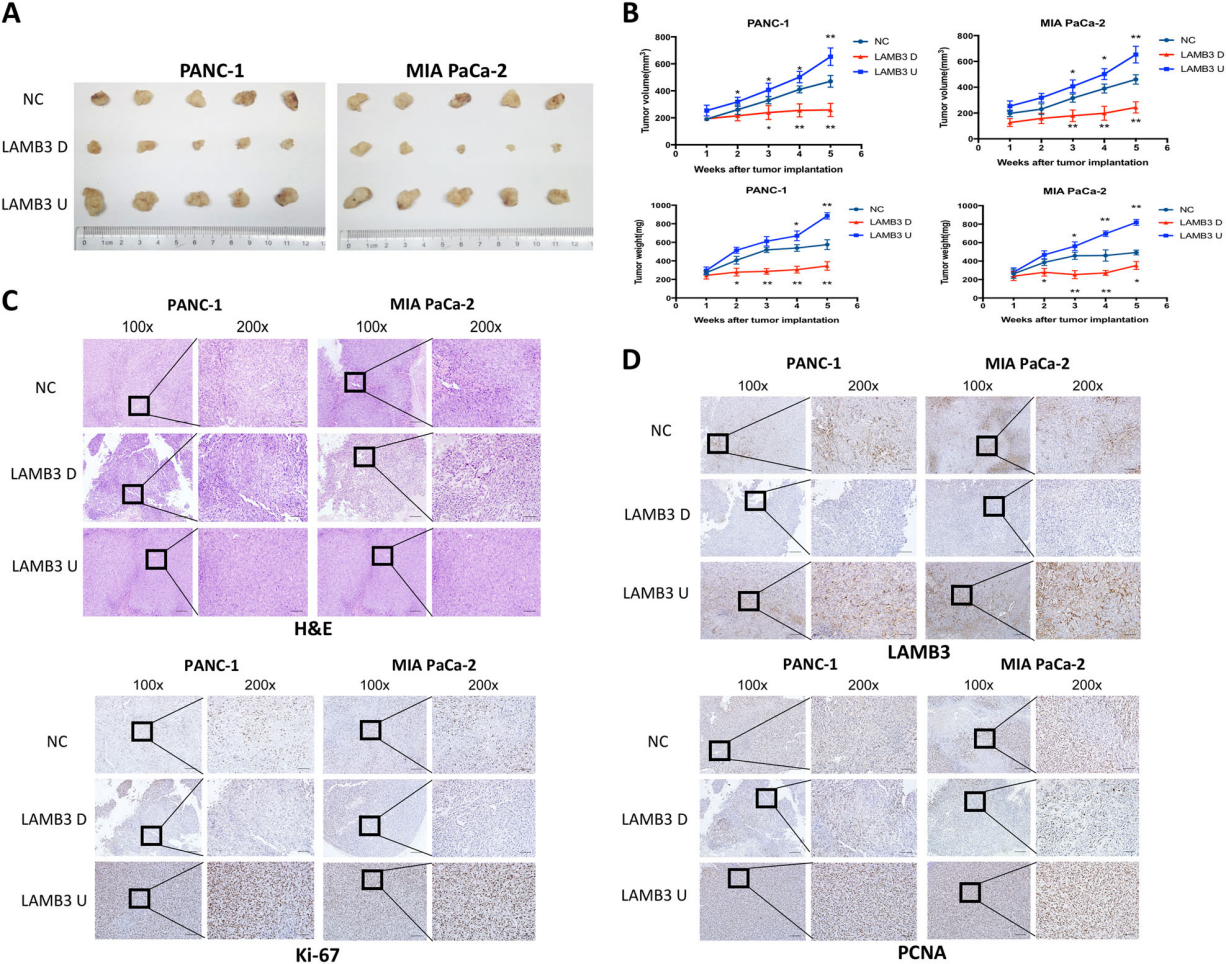
LAMB3 promotes pancreatic cancer cell proliferation in vivo. **a** Images of subcutaneous xenografts from mice in the LAMB3U, LAMB3D, and NC groups. *N* = 5. **b** Tumor volume growth curves and tumor weight change curves for subcutaneous xenografts. **c** Representative images of H&E-stained xenograft tumor sections from BALB/c nude mice subcutaneously injected with PANC-1 or MIA PaCa-2 LAMB3U, LAMB3D, or NC cells (scale bars: 100 μm (left) and 50 μm (right)). **d** The expression of LAMB3 and the proliferation markers Ki-67 and PCNA was examined in xenograft tumor tissue sections using immunohistochemistry (IHC; scale bars: 100, 50, and 20 μm from left to right). (*P < 0.05, **P < 0.01)

To determine whether LAMB3 plays a role in PDAC proliferation in vivo, a subcutaneous xenograft model of PANC-1 and MIA PaCa-2 cells in Balb/c nude mice was established. The LAMB3 knockdown group showed a slower increase in tumor volume and weight than did the control group (Fig. 8a, b). H&E staining of serial tumor sections from the mouse xenograft model is shown in Fig. 8c, and LAMB3, Ki-67, and PCNA IHC was significantly reduced in the LAMB3 knockdown group compared with the NC group (Fig. 8d); the opposite effects were observed in the LAMB3 overexpression group.

### LAMB3 promotes pancreatic cancer cell invasion and migration in vivo

**Fig. 9:**
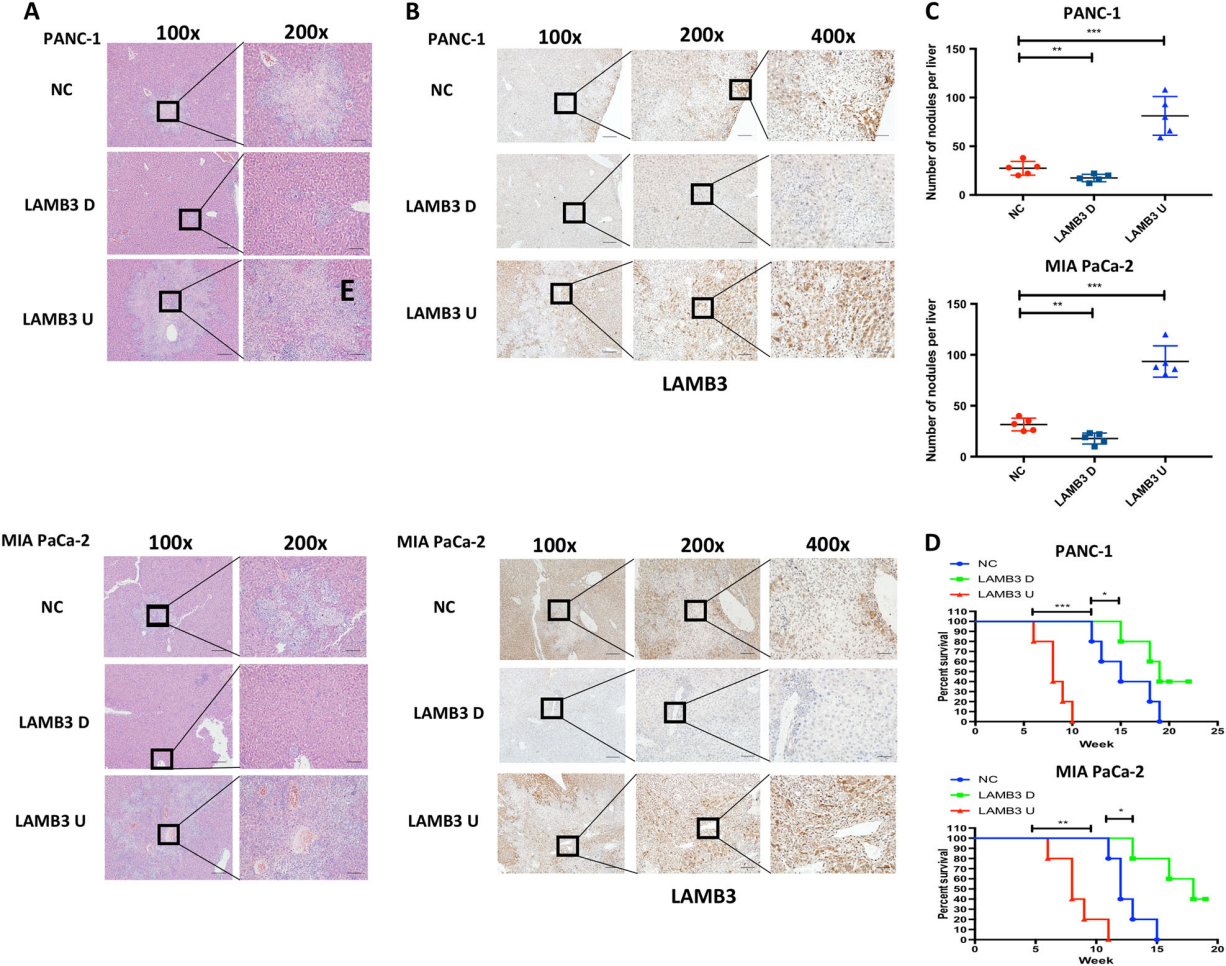
LAMB3 promotes pancreatic cancer cell invasion and migration in vivo. **a** Representative H&E-stained images of liver tissue sections from BALB/c nude mice injected with PANC-1 or MIA PaCa-2 LAMB3U, LAMB3D, or NC cells (scale bars: 100 μm (left) and 50 μm (right)). **b** LAMB3 expression was determined in representative metastatic liver tumor foci sections from the xenografts using IHC (scale bars: 100, 50, and 20 μm from left to right). **c** The number of metastatic liver nodules in each group was observed (*n* = 5, **P < 0.01, ***P < 0.001). **d** Kaplan–Meier analysis of the overall survival rate for each group (*n* = 5, *P < 0.05, **P < 0.01, ***P < 0.001)

A subcutaneous xenograft model and a model in which intrasplenically injected human PDAC cells induce hepatic metastasis were generated. In the xenograft model, mice were monitored and weighed weekly and then euthanized in the sixth week. There was no significant difference in tumor growth and weight in the NC, LAMB3 overexpression, and LAMB3 knockdown groups. H&E staining of whole-xenograft serial sections indicated that mice injected with LAMB3-overexpressing cells had more metastatic foci than did control mice (Fig. 9a). LAMB3 IHC staining was significantly reduced in the LAMB3 knockdown group compared with the control group (Fig. 9b). Survival curves and gross pathologic analyses showed that LAMB3 knockdown decreased the number of liver metastatic nodules and prolonged the overall survival compared with the NC, while LAMB3 overexpression had the opposite effects (Fig. 9c, d).

## Discussion

Many recent studies have indicated that adhesion and extracellular matrix proteins contribute to the progression of a variety of solid tumors, including pancreatic cancer^20–22^. LM-332, which is encoded by LAMA3, LAMB3, and LAMC2, is related to tumor invasiveness in various types of malignant tumors^23–25^. LAMB3 is a potential biomarker of cancer invasion and metastasis that is involved in the focal adhesion pathway, but a role for LAMB3 in pancreatic cancer has not been investigated previously^26,27^.

In this study, we first evaluated LAMB3 expression in human PDAC and found it to be significantly higher in all PDAC tissues than in adjacent normal tissues. Clinical data from our retrospective study and the GSE and TCGA datasets also showed that LAMB3 overexpression was significantly associated with tumorigenesis and TNM stage in PDAC (Supplementary Table 1). In addition, we selected PANC-1 and MIA PaCa-2 cells (higher LAMB3 mRNA and protein expression) to conduct the following experiments. LAMB3 knockdown by short hairpin RNA (shRNA) had a significant effect on the proliferation of these two cell lines, as shown by CCK-8 and colony formation assays. In addition, the invasion and migration of PANC-1 and MIA PaCa-2 cells in transwell and wound healing assays were considerably reduced by LAMB3 knockdown, while LAMB3 overexpression had the opposite effects.

EMT is a process by which epithelial cells lose their polarity and adhesion after undergoing molecular reprogramming and phenotypic changes, and gain migratory and invasive properties to become mesenchymal stem cells^28–30^. Our GSE analysis showed that LAMB3 overexpression was significantly related to cell adhesion in PDAC. LAMB3 knockdown by shRNA significantly reduced p-Akt-S473 levels without altering total Akt protein levels, indicating that LAMB3 could promote tumor invasion by activating the Akt signaling pathway in PDAC. Increased Akt activity and Snail stabilization are essential for EMT. As a result, the expression of some proteins downstream of the Akt pathway, such as p53, BCL-2, and cyclin D1, which are involved in cell survival, antiapoptosis signaling and cell cycle arrest, respectively, were also influenced. MMPs are considered critical mediators of cellular events, including cell proliferation, apoptosis, invasion, migration, and morphological changes^31–33^. Akt activation has been shown to induce EMT and MMP9 activity and to promote tumor invasiveness and metastasis^34,35^. Our analysis confirmed that LAMB3 regulates the EMT-related proteins E-cadherin, N-cadherin, Snail, Slug, vimentin, MMP2, and MMP9 via the activation of Akt signaling.

Our GSE analysis also showed that LAMB3 overexpression was positively correlated with the regulation of the phosphatidylinositol signaling system in PDAC. The PI3K/Akt signaling pathway is one of the best studied pathways in the phosphatidylinositol signaling system, which plays a crucial role in regulating normal cellular processes involved in tumorigenesis, cell growth, proliferation, metabolism, motility, survival, and apoptosis.

Aberrant activation of the PI3K/Akt signaling pathway promotes the survival and proliferation of tumor cells in many human cancers^36–38^. Akt is partially activated by T308 phosphorylation by PDK1. Full activation requires S473 phosphorylation, which can be catalyzed by multiple proteins, including PDK2^39,40^. To investigate the upstream component that mediates Akt regulation by LAMB3, we focused on PI3K and PDK2. In our study, the PI3K/Akt pathway inhibitor LY294002 also significantly decreased the levels of p-Akt-S473, N-cadherin, Snail, and Slug in both the NC and LAMB3-overexpressing groups, whereas E-cadherin levels were significantly increased in only LAMB3-overexpressing MIA PaCa-2 cells. Total Akt levels were not significantly affected. These results further indicated that LAMB3-mediated invasion and migration may occur via the PI3K/Akt signaling pathway. Finally, we have shown that LAMB3 regulates the invasive and metastatic potential of PDAC in vivo by mouse tumor xenograft and liver metastasis models.

In summary, we demonstrated that LAMB3 promotes PDAC cell invasion and migration by activating the PI3K/Akt signaling pathway in vitro and in vivo. Our present results showed that LAMB3 is upregulated in PDAC tissues, and high LAMB3 expression is linked to processes involved in tumorigenesis, such as apoptosis, cell cycle, proliferation, migration and invasion, by activating the PI3K/Akt signaling pathway in PDAC. To our knowledge, this may be the first study to reveal that LAMB3 is related to the occurrence and development of PDAC and to elucidate the relevant molecular mechanism of LAMB3 in PDAC. At present, many inhibitors of the PI3K/Akt signaling pathway have been developed, such as LY294002, PX-866, BKM120, SAR245408, XL147, and GDC-0941^41,42^. Our study may provide a potential target for pharmaceutical development and novel therapeutic strategies for the use of these PI3K/Akt pathway inhibitors to control PDAC invasiveness and metastasis in the future. Furthermore, we found that PDAC patients with high LAMB3 expression presented with a relatively more advanced TNM stage. Thus, high LAMB3 expression may be an independent prognostic factor in patients with resected pancreatic carcinoma and a new clinical indicator for choosing the appropriate individualized treatment method^43^.

## Materials and methods

### Cell culture and reagents

The human pancreatic cancer cell lines PANC-1 and MIA PaCa-2 were purchased from ATCC and grown in Dulbecco's Modified Eagle Medium (DMEM) supplemented with 10% heat-inactivated fetal bovine serum (FBS; Gibco, CA, USA). All cells were maintained in a standard humidified incubator (37 °C, 5% CO_2_). LY294002 was purchased from Cell Signaling Technology, Inc. (MA, USA).

### Cell proliferation assay

**Fig. 10:**
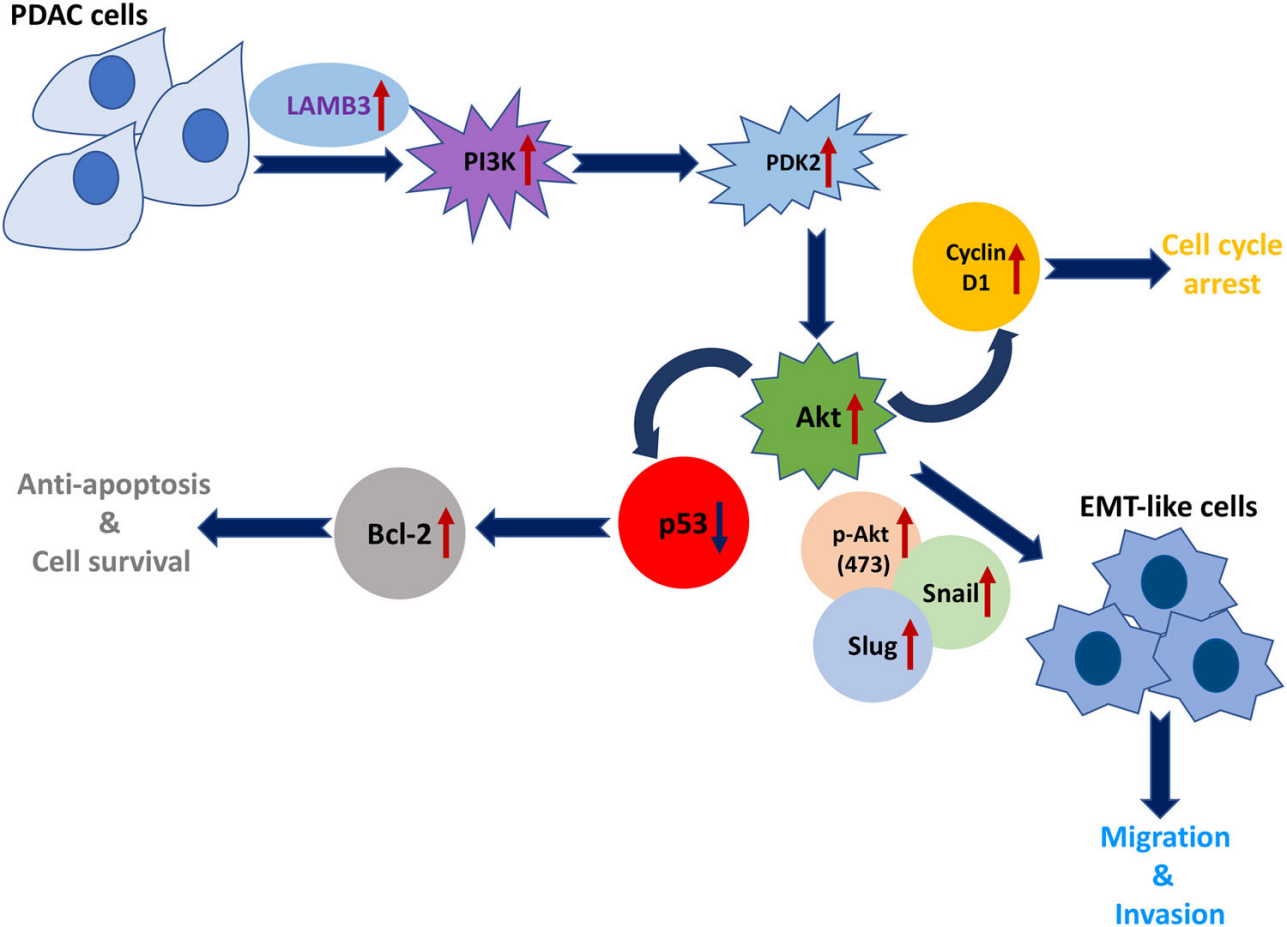
**The proposed mechanism of LAMB3-mediated tumor antiapoptotic signaling, invasion and metastasis through the PI3K/Akt axis in patients with pancreatic ductal adenocarcinoma (PDAC)**

PANC-1 and MIA PaCa-2 cells (3 × 10^3 ^cells/well) were seeded in 96-well plates overnight. All cells were transfected with small interfering RNA (siRNA) on the next day. CCK-8 solution (Abcam, MA, USA) was added to each well. After 2 h of incubation, the plates were shaken, and the absorbance was measured using a SpectraMax M5 plate reader (Molecular Devices, Sunnyvale, CA, USA) at 450 nm (OD450). The same procedure was repeated every 24 h until the last plate had been assayed. All assays were repeated in triplicate (Fig. 10).

### Wound healing assay

PANC-1 and MIA PaCa-2 cells (1 × 10^6^ cells/well) were cultured in 6-well plates. A wound was generated in confluent cell monolayers by standardized wound scratching using a sterile 200 μl pipette tip, and the cells were then incubated in culture medium with 1% FBS and 5 ng/ml TGF-β. Cell migration into the wound area and recovery of the monolayer were monitored (0–72 h) by a phase contrast microscope and digitally photographed (Nikon Diaphot 300; Nikon, Tokyo, Japan).

### Cell invasion and migration assays

Transwell membranes (Corning, NY, USA) were coated with Matrigel for 6 h for invasion assays and used without Matrigel for migration assays. PANC-1 and MIA PaCa-2 cells (0.05 × 10^6^) in serum-free medium were seeded into the upper chambers, and 600 μl of medium supplemented with 10% FBS was added to the lower chambers. After incubation for 24 h, the cells adhering to the upper surface of the membrane were removed. Meanwhile, the invaded or migrated cells, which adhered to the lower surface, were stained with 0.1% crystal violet and measured by optical microscopy.

### Real‑time quantitative polymerase chain reaction (qPCR)

Total RNA was extracted from PANC-1 and MIA PaCa-2 cells using TRIzol reagent (Invitrogen, CA, USA), reverse transcribed and amplified using primers for LAMB3 and glyceraldehyde 3-phosphate dehydrogenase (GAPDH). The RT‑PCR primer sequences (Invitrogen, CA, USA) were LAMB3 (forward) 5′‑CCAAAGGTGCGACTGCAATG‑3′ and (reverse) 5′‑AGTTCTTGCCTTCGGTGTGG‑3′ and GAPDH (forward) 5′‑ACAACTTTGGTATCGTGGAAGG‑3′ and (reverse) 5′‑GCCATCACGCCACAGTTTC‑3′.

### Colony formation assay

Cells were seeded at an initial density of 500 cells/well and cultured in six-well plates. After 2 weeks of cultivation, the cells were fixed with 4% paraformaldehyde and stained with 0.1% crystal violet (Sigma, MO, USA). Colonies containing over 50 cells were counted using a light microscope.

### Immunofluorescence

Cells (0.02 × 10^6^) were seeded on glass cover slips (Thermo Fisher Scientific, MA, USA) and cultured in a standard humidified incubator (37 °C, 5% CO_2_). The cells were fixed with 500 µl of 4% paraformaldehyde for 10 min, permeabilized in 0.2% Triton X-100 for 5 min and blocked with 5% BSA for 30 min at room temperature. Primary antibodies against p-Akt-S473 (Cell Signaling Technology, MA, USA) and LAMB3 (Abcam, MA, USA) were added to the slides at a 1:100 dilution in 5% BSA, and the slides were incubated overnight in a moist box in the cold room; Phosphate-Buffered Saline (PBS) was added to the NC slides. The next morning, Cy3- and Cy5-labeled secondary antibodies (Thermo Fisher Scientific, MA, USA) were added at a 1:200 dilution in PBS, and the slides were incubated for 1 h at room temperature in the dark. All washes were performed with 1 × PBS. The slides were mounted in antifade solution containing DAPI (Thermo Fisher Scientific, MA, USA). Images were taken with a Leica TCS SP8 confocal microscope (Leica, IL, USA).

### Western blot analysis

Cells were lysed in lysis buffer containing 50 mM HEPES, pH 8.0, 10% glycerol, 1% Triton X-100, 150 mM NaCl, 1 mM EDTA, 1.5 mM MgCl_2_, 100 mM NaF, 10 mM Na_4_P_2_O_7_·10H_2_O, and a protease inhibitor cocktail (Roche Applied Science, Mannheim, Germany). Frozen tissue samples stored in liquid nitrogen were cut into pieces with scissors. Each sample was homogenized in lysis buffer at a ratio of 1:20. After centrifugation at 14,000 rpm for 20 min, the supernatant was collected. A BCA Protein Assay kit (Thermo Scientific, IL, USA) was used to measure total protein concentration. Aliquots (50 μg) of total cellular protein were resolved by sodium dodecyl sulfate polyacrylamide gel electrophoresis (6–15%) and electrotransferred onto Polyvinylidene Fluoride (PVDF) membranes, which were blocked with 5% skim milk at room temperature for 1 h. The membranes were then incubated with primary antibodies against the following proteins for western blot analysis: LAMB3, E-cadherin, and N-cadherin (1:1000; Abcam, MA, USA); phospho-Akt-S473, total Akt, vimentin, Slug, Snail, and β-actin (1:1,000; Cell Signaling Technology, MA, USA); and LAMA3 and LAMC2 (1:1000; Santa Cruz Biotechnology, Santa Cruz, CA, USA). Following incubation with the corresponding secondary antibodies (1:5000; GE Healthcare, Buckinghamshire, UK), immune reactive bands were visualized by enhanced chemiluminescence (ECL) detection.

### IHC staining and TMAs

TMAs were created by Shanghai Outdo Biotechnology Co., Ltd. (Shanghai, China). IHC staining with antibodies against LAMB3, Ki-67, and PCNA (Abcam, MA, USA) was performed to detect protein expression levels following standard operating procedures. The positive staining scores were calculated by multiplying the percentage positive (0, < 5% of cells; 1, 5–25%; 2, 26–50%; 3, 51–75%; and 4, 76–100%) by the staining intensity score (0, no coloration; 1, pale yellow; 2, yellow; and 3, dark brown) and were classified as follows: negative (0, −); weakly positive (1–3, + ); moderately positive (4–8, + + ); and strongly positive ( > 8, + + + ). We divided all patients into two groups (−/ + , low expression; and + + / + + + , high expression) and performed survival analyses.

### Plasmid and lentivirus production

We used the lentiviral vector pLKO.1-puro-CMV-TurboGFP (VT8114, Youbio, Changsha, China) to knock down LAMB3 expression (LAMB3D). The sequence of the 21-nucleotide shRNA targeting LAMB3 was GAAGCTTCAATGGTCTCCTTA. LAMB3 was overexpressed (LAMB3U) using pCDH-CMV-MCS-EF1-puro (VT1480, Youbio, Changsha, China). Knockdown or overexpression lentivirus particles were generated by cotransfecting lentivirus constructs with pMD2.G (plasmid 12259, Addgene, Cambridge, MA, USA) and psPAX2 (plasmid 12260, Addgene, Cambridge, MA, USA) at a 4:3:1 ratio into HEK-293T cells. The pCDH-CMV-MCS-EF1-puro empty vector was used as a NC.

### Tumor xenograft model

Female 6-week-old BALB/c nude mice were provided by the Experimental Animal Study Center of Hubei Province. All mice were bred under specific pathogen-free conditions in the Experimental Animal Center of the Wuhan Institute of Virology, CAS. Female age-matched mice were used in all experiments. A total of 1 × 10^7^ transfected cells were subcutaneously injected into the left armpit of the mice (*n* = 5 per group). Mouse weight and tumor diameter were measured every week. Mice were sacrificed 6 weeks after the initiation of treatment, and the tumor weight and volume were evaluated.

### Pancreatic cancer liver metastasis model

Human pancreatic cancer cells (5 × 10^6^) were resuspended in serum-free medium and injected into the involucrum of the spleen of female 8-week-old BALB/c nude mice (*n* = 5 per group). Splenectomy was performed 30 min later. Mice were weighed once a week and sacrificed when cachexia was observed or if the mice exhibited > 15% weight loss. Mouse livers were subsequently evaluated both macroscopically and microscopically.

### Statistical analysis

All experiments were performed in triplicate and were independently conducted three times. GraphPad Prism 7.0 (GraphPad Software, USA) was used for the statistical analyses. Data are presented as the mean ± SD. Statistical significance was determined using Student’s *t*-test for the two groups and one-way ANOVA for multiple groups. Significant differences between two mean values were estimated using Student’s *t*-test. A *P*-value < 0.05 was considered to indicate statistical significance.

## Supplementary information

Supplementary table 1
