# Supplementary material for: LAMB3 mediates apoptotic, proliferative, invasive, and metastatic behaviors in pancreatic cancer by regulating the PI3K/Akt signaling pathway
Source: Cell Death Dis. 2019 Mar 8;10(3):230. doi: 10.1038/s41419-019-1320-z (PMC6408539; doi:10.1038/s41419-019-1320-z)
Supplement: Supplementary file 1 — Supplementary table 1 [file 41419_2019_1320_MOESM1_ESM.docx]

11(25.00%)

10(50%)

10(50%)

8(16.67%)

0.0002

***

*P*

0.1545

0.7178

0.0687

0.3806

0.6657

0.1895

**LAMB3 expression**

**Differentiation status**

**Table 1 The correlation between clinicopathological parameters and LAMB3 expression**

Low, *n*(%)

High, *n*(%)

**Age**

≤ 50

> 50

**Gender**

Male

Female

**Alcohol consumption**

Ever and current

Never

**Smoking status**

Ever and current

Never

**Tumor size**

<5 cm

≥5 cm

Well or Moderate

Poor

**TNM**

I–II

III

27(32.92%)

55(67.08%)

22(34.92%)

41(65.08%)

15(38.46%)

24(61.54%)

13(27.08%)

35(72.92%)

24(44.44%)

30(55.56%)

16(33.33%)

33(75.00%)

32(66.67%)

20(34.48%)

38(65.52%)

17(38.64%)

27(61.36%)

28(52.83%)

40(83.33%)

25(47.17%)

13(46.43%)

24(32.43%)

50(67.57%)

15(53.57%)
